# The State of Vaccine Confidence in Poland: A 2019 Nationwide Cross-Sectional Survey

**DOI:** 10.3390/ijerph17124565

**Published:** 2020-06-24

**Authors:** Filip M. Furman, Wojciech Stefan Zgliczyński, Mateusz Jankowski, Tomasz Baran, Łukasz Szumowski, Jarosław Pinkas

**Affiliations:** 1 Home Hospice “Socrates”, Armii Krajowej 2/4, 05-800 Pruszków, Poland; filip.furman@gmail.com; 2 School of Public Health, Centre of Postgraduate Medical Education, Kleczewska 61/63, 01-826 Warsaw, Poland; mjankowski@cmkp.edu.pl (M.J.); jpinkas@cmkp.edu.pl (J.P.); 3 Faculty of Psychology, University of Warsaw, Stawki 5/7, 00-183 Warsaw, Poland; tomasz.baran@psych.uw.edu.pl; 4 Department of Cardiac Arrhythmia, National Institute of Cardiology, Alpejska 42, 04-628 Warsaw, Poland; lszumowski@ikard.pl

**Keywords:** vaccination, vaccines, trust, vaccine hesitancy, vaccine trust, Poland

## Abstract

Vaccination is considered as one of the most successful and cost-effective public health interventions. This study aimed to assess (1) the attitudes and behaviors towards mandatory childhood vaccination, with particular emphasis on socio-economic factors determining the vaccine confidence among adults in Poland as well as to (2) identify the potential impact of anti-vaccination movement on vaccination coverage among children and adolescents aged ≤19 years. This cross-sectional study was carried in 2019 on a nationwide, representative sample of 1079 individuals aged 18 and over in Poland (53.7% females). Most of the respondents (74.6%) agreed or strongly agreed that mandatory vaccinations are safe, and only 8% of participants neglected the safety of vaccines. The results of multivariate analysis showed that the lowest level of vaccine confidence was observed among participants aged 25–34 years (aOR: 0.48, 95%CI: 0.29–0.80; *p* = 0.01). There was a positive correlation (*r* = 0.35; *p* < 0.001) between trust in doctors and vaccine confidence. Moreover, there was a positive correlation between trust in scientific knowledge and vaccine confidence (*r* = 0.19; *p* < 0.001). Also, a negative correlation (*r* = −0.13; *p* < 0.001) between trust in horoscopes and vaccine confidence was observed. Most of the parents declared (97.7%), that their children were vaccinated following the national immunization programme. However, 8.5% of parents who currently vaccinated their children declared that they would stop vaccinating children when vaccination obligation will be abolished. This study demonstrates relatively high confidence in mandatory vaccination among adults in Poland. While most of society trusts in vaccine safety, young adults are the least trustful of vaccinations.

## 1. Introduction

Vaccination is considered as one of the most successful and cost-effective public health interventions [1,2,3]. According to the World Health Organization (WHO) estimates, immunization prevents up to 3 million deaths each year [3]. Global coverage with vaccines to prevent diphtheria-tetanus-pertussis, tuberculosis, poliomyelitis and measles has increased from less than 5% in 1974 to 86% in 2018 [4]. The WHO Global Vaccine Action Plan 2011–2020 calls on all the countries to provide equitable access to existing vaccines for people in all communities [5]. Vaccination policies, including the extent of the immunization programme, obligation or voluntary nature, and funding source, varies across the countries [6,7]. In the 20th century, Poland as well as other post-war communist countries from Central and Eastern Europe (CEE) introduced mandatory vaccinations [8]. In Poland, mandatory vaccinations were maintained also after the collapse of the communist regime in 1989 [8,9].

The Constitution of the Republic of Poland states that “public authorities are obliged to combat epidemic diseases” [9]. This provision referring to infections and infectious diseases in humans makes public authorities responsible for not only elimination of outbreaks of disease, but also for preventative measures to counteract diseases. In accordance with Polish law, children and adolescents up to 19 years are subject to the vaccination obligation [9]. Mandatory vaccinations are free of charge. The national immunization schedule is annually updated by the Chief Sanitary Inspector decree [10,11]. This announcement specifies the infectious diseases against which preventative vaccinations are performed and the age of people who are subject to the vaccination. In 2019, 11 vaccines, including those against tuberculosis, hepatitis B, diphtheria, pertussis, tetanus, Haemophilus influenzae type b, pneumococcal disease, poliomyelitis, measles, mumps, rubella were mandatory [11]. Moreover, there are some other vaccines, which are recommended but not free of charge [11]. Parents or carers are obligated to vaccinate their children following the current national immunization schedule [9,11]. Family doctors are responsible for checking whether their patients have had all the mandatory vaccines. Refusal to vaccinate from non-medical reasons is subject to a fine [9,11]. Moreover, in the case of notorious refuse of vaccination, which may pose a threat to the life or health of the child, the applicable provisions even allow for the limitation or withdrawal of the parental authority of parents who do not comply with the obligation to vaccinate their children [11].

In recent years, there has been a growing number of parents who refuse immunizations for their children [12]. For example, in Poland between 2012 and 2018, the number of parents who refused immunizations of their children increased by over seven times (from 5340 in 2012 to 40 342 in 2018) [12]. The WHO has identified vaccine hesitancy as one of the top ten global health threats of 2019 [13]. In recent years, there was a rise in the anti-vaccination movement in Europe. In 2018, the anti-vaccination movement attempted to abolish mandatory childhood vaccination in Poland [14]. The public bill was submitted to the Polish parliament on the initiative of the association related to the anti-vaccination movement. Parliamentary work was accompanied by a wide media discussion on preventive vaccinations. Public health institutions, medical authorities and chambers of physicians were actively involved in the public debate to present evidence-based knowledge about vaccinations [14,15]. Nevertheless, some media and websites (including web television) actively promoted the theories presented by the leaders of the anti-vaccination movement. Finally, the project was rejected and the vaccination obligation was maintained [14]. However, the influence of this debate on public attitudes towards vaccination and vaccine trust is unknown. Moreover, the potential impact of the anti-vaccination movement on refusals to vaccinate among Poles and vaccine hesitancy among parents has not been thoroughly studied. Therefore, this study aimed to assess (1) the attitudes and behaviors towards mandatory childhood vaccination, with particular emphasis on socio-economic factors determining the vaccine confidence among adults in Poland as well as to (2) identify the potential impact of the anti-vaccination movement on vaccination coverage among children and adolescents aged ≤19 years.

## 2. Materials and Methods

### 2.1. Study Design and Population

This cross-sectional study was carried out between 15 and 18 March 2019 on a nationwide, representative sample of 1079 individuals aged 18 and over in Poland. The computer-assisted web interview (CAWI) technique was used [16]. All the interviews were carried out by a specialized survey company—“Ariadna” National Research Panel (Warsaw, Poland) [17]—on behalf of the research team, which provides the context of this research. The operational number of the “Ariadna research panel” is over 110,000 registered and verified individuals aged 15 and over [17]. A two-step non-probability quota sampling was applied. In the first step, the population was segmented into subgroups based on mutual exclusivity. Then, from such separated groups, respondents were selected based on the stratification model, including gender, age as well as the size of domicile and the territorial distribution within voivodships across Poland [18]. The stratification was based on demographic data from the Central Statistical Office (Warsaw, Poland) [18]. A quota sample used for this survey ensured that the sample structure corresponds with the structure of the population.

Participation in the study was voluntary and anonymous. The study protocol was reviewed and approved by the Ethical Review Board at the Centre of Postgraduate Medical Education, Warsaw, Poland (consent number: 48/PB/2020).

### 2.2. Study Questionnaire

The research tool was a questionnaire developed for the purpose of this study. In preparation of the questionnaire, we analysed the previously published nation-wide cross-sectional surveys about knowledge, beliefs, and attitudes towards vaccinations, with special emphasis on the EU report on vaccine confidence [19,20]. The questionnaire included 12 questions related to attitudes and behaviors towards mandatory childhood vaccinations. Questions also addressed personal characteristics, including gender (male or female), age (years), marital status (ever/never married), having children, educational level, occupational status (including professional activity) and place of residence (rural or urban). In the case of the age criterion, the following was applied: 18–24 years, 25–34 years, 35–44 years, 45–54 years, and 65+ years. Educational level was classified as higher education or less than higher (primary, vocational or secondary). The occupational activity was classified as active (currently employed) or passive (currently unemployed, students or retires) occupational status.

Beliefs and attitudes towards vaccinations were assessed according to the answers to the question “How much do you agree with the following statement”, with six items: (1) mandatory vaccinations (vaccines) are safe; (2) doctors can be trusted; (3) I trust my doctor; (4) only scientific knowledge is trustworthy; (5) horoscopes (astrological signs) contain a lot of truth; (6) governments usually want the well-being of citizens. All 6 questions were meant to assess attitudes and beliefs with a 5-point response scale: 1 = “strongly disagree”, 2 = “tend to disagree”, 3 = “hard to say/undecided”, 4 = “tend to agree” and 5 = “strongly agree”. An additional question regarding the childhood vaccination was addressed to all participants who declared having children: “Has your child been vaccinated (applies to mandatory childhood vaccination listed in the national immunization schedule)/Have you ever refused (even once) to vaccinate your child despite the absence of medical contraindications?” and “Would you vaccinate your children in case of abolition of the obligation to vaccinate?” (Yes/No). In this study, vaccine confidence was measured through perceived vaccine safety, which is justified by the fact that the main argument of the anti-vaccination movement in Poland was to undermine the safety of vaccinations.

### 2.3. Statistical Analysis

The data were analysed with SPSS version 25 software (IBM, Armonk, NY, USA). The distribution of categorical variables was shown by frequencies and proportions (with 95% confidence interval—95%CI). A chi-square test was used to compare categorical variables. To investigate the relationship between vaccine confidence and trust in doctors and medicine, scientific knowledge and the government, a Spearman correlation was performed. The univariable and multiple logistic regression analysis were implemented to calculate the odds ratios (ORs) and the 95% confidence interval (CI) of selected variables in relation to the (1) vaccine confidence and (2) willingness to discontinue of vaccination in the future. Statistical inference was based on the criterion *p* < 0.05.

Associations between personal characteristics (gender, age, marital status and having children), socio-economic characteristics (educational level, occupational status) and housing characteristics (place of residence) with vaccine confidence (defined as a trust in vaccine safety: “tend to agree” or “strongly agree”) were conducted using the logistic regression analyses. Model one assessed the impact of personal characteristics on the level of vaccine confidence. Model two adds socio-economic variables. Model three adds place of residence.

Similar linear regression models were constructed for the outcome of discontinuation of childhood vaccination in case of abolition of the obligation to vaccinate.

## 3. Results

The analysis is based on responses to survey forms received from 1079 individuals (53.7% females; at the age of 18 to 88 years). Table 1 shows the characteristics of the sample classified by the state of vaccine confidence. Most of the respondents (74.6%) agreed or strongly agreed that mandatory vaccinations are safe, and only 8% of participants neglected the safety of vaccines. The highest proportion of participants (12%) that undermines the safety of vaccinations was observed among those aged 25–34 years (*p* = 0.04). There was no influence (*p* > 0.05) of factors such as gender, marital status, having children, educational level, occupational status, type of professional activity and place of residence on the state of vaccine confidence among Poles.

The results of multivariate analysis (Table 2) confirmed that the lowest level of vaccine confidence was observed among participants aged 25–34 years (aOR: 0.48, 95%CI: 0.29–0.80; *p* = 0.01).

**Figure 1:**
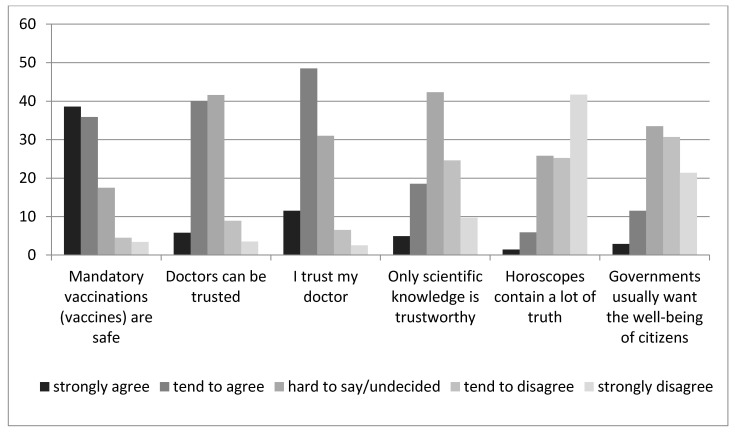
Participants’ responses to the question ‘How much do you agree or disagree with the following statement?’, Poland, 2019 (*n* = 1079).

Among the participants, 60% trusted their doctor, and 45.9% believed that, overall, doctors can be trusted (Figure 1). However, less than every fourth respondent (23.4%) declared that only scientific knowledge is trustworthy. There was a positive correlation between trust in doctors and vaccine confidence (Table 3). Moreover, there was a positive correlation between trust in scientific knowledge and vaccine confidence. Also, a negative correlation between trust in horoscopes and vaccine confidence was observed (Table 3).

Most of the parents declared (97.7%), that their children were vaccinated following the national immunization programme (without significant differences by personal characteristics or socio-economic factors). However, 8.5% of parents who currently vaccinated their children declared that they would refuse the vaccination for their children in the case of abolition of the obligation to vaccinate in Poland. There was no influence (*p* >0.05) of gender, age, marital status, educational level, occupational status, place of residence on the parents’ attitudes towards the vaccination of their children (Table 4).

## 4. Discussion

To the authors’ best knowledge, this is the most up-to-date study on the state of vaccine confidence among adults in Poland, carried out after the parliamentary debate about the public’ bill that aimed to abolish compulsory vaccinations for children. In our study, the lowest level of vaccine confidence was observed among adults aged 25–34 years. Moreover, vaccine confidence was positively correlated with trust in medicine, doctors or scientific knowledge, and negatively correlated with trust in horoscopes. The present findings also point to the need to maintain vaccination obligations. While only 2.3% of parents declared that they have avoided mandatory childhood vaccination at least once, 8.5% of parents would stop vaccinating their children if the vaccination obligation was abolished.

The state of vaccine confidence differs between the countries [19,20]. Results of the Vaccine Confidence Project (VCP)—one of the largest cross-sectional surveys on vaccine confidence (65,819 individuals across 67 countries; 2015) showed that the European region had lower confidence in the safety of vaccines than other world regions [20]. In 2015, among the European Union (EU) countries, the lowest levels of safety-based confidence issues were observed in France, Greece, Slovenia, and Italy [19]. In 2018, a similar study was carried out among 28,782 participants from 28 EU countries. The majority of the EU citizens agreed that vaccines are important (90.0%), safe (82.8%) and effective (87.8%) [19]. However, attitudes towards vaccination among EU citizens changed between 2015 and 2018 [19]. In 2015–2018, the state of vaccine confidence increased in France, Greece, Slovenia and Italy, but decreased in Poland, the Czech Republic, Finland and Sweden [19]. Over 4 years in Poland, the percentage of respondents agreeing that vaccines are important for children has decreased by 9.2%; vaccines are safe by 7.3%; vaccines are effective by 7.7% [19]. Overall in 2018, 75.9% of Poles declared that it is important to vaccinate children, 72.4% trusted in vaccine safety and 74.9% agreed that vaccines are effective [19]. The opposite trend was observed in the study (two waves, 2017 and 2018) by the Centre for Public Opinion Research (CBOS) [21]. Between 2017 and 2018, the proportion of Poles who trusted in vaccine safety increased from 73% to 86% [21]. Moreover, the proportion of Poles who agreed with the statement that vaccination is the most effective way of protecting children against serious diseases increased from 86% in 2017 to 93% in 2018 [21].

The present findings also support the hypothesis that vaccine confidence in Poland is growing. Compared to the EU report from 2018 [19], the proportion of Poles who trust in vaccine safety increased by 2.2%. The percentage of people who negate the safety of vaccinations in our study (7.9%) is comparable to that observed in the CBOS 2018 survey (5%) [21]. In our study, 17.5% of Poles were undecided when asked about vaccination safety, which is two times higher compared to the CBOS survey (9%) [21]. This finding indicates a further need for education on vaccine safety to convince the undecided groups.

Previous studies reported a variety of socio-demographic factors related to attitudes towards vaccinations such as gender, age, ethnicity, educational level, income class [22,23,24]. Moreover, social attitudes towards vaccination may be grounded in the cultural, religious, historical and geopolitical context of each country [24,25,26]. In Central and Eastern Europe, attitudes towards vaccination may be partly shaped by past experience of the communist period and the organization of the healthcare system prior to 1989. The present findings point that age was the only factor that significantly shaped trust in vaccine safety among Poles. The lowest level of vaccine confidence was observed in the group aged 25–34. This age group is predominantly people who start families and decide to have their first child. In addition, people born in the 90s are a generation with broad access to the Internet, which has been present on the Internet and social media since theywere a young age. The Internet and social media are the main communication channels for the anti-vaccination movement, so people aged 25–34 who search for health information on the internet can be an easy target for the anti-vaccination movement. A systematic review by Fournet et al. showed that educational level may have an impact on beliefs, attitudes and reasons for non-vaccination [24]. In our study, the largest number of opponents of vaccinations are people with higher education, but this observation was not statistically significant (*p* = 0.05).

The public bill that aimed to abolish compulsory vaccinations for children was submitted to the Polish parliament in 2018 [14,15]. The anti-vaccination movement built a base of support for the collection of 100,000 signatures necessary to submit a legislative proposal to the Polish Parliament [15]. The Polish anti-vaccination movement originated from a small group of parents who created a hierarchical organization that chose social media as the main communication channel. Anti-vaccine leaders have set up a dedicated website and groups on popular social networks such as Facebook to promote their theories. Moreover, they posted live streams and videos on Facebook and YouTube. Due to the lack of scientific basis for the content of the anti-vaccination movement, they were overlooked by major media in Poland. Nevertheless, the representatives of the anti-vaccination movement were often invited to web television (web TV). Broad access to the Internet meant that anti-vaccination theories were targeted at recipients of this type of web-based media [27]. Social media and Internet television can be a public health communication channel that will reach young adults—a group with the lowest level of vaccine confidence.

Despite the growing number of people who get health information from the internet, healthcare professionals remain the most trusted advisors and influencers of vaccination decisions [28]. In our study, vaccine confidence was positively correlated with trust in medicine, doctors or scientific knowledge, and negatively correlated with trust in horoscopes. The present findings point to the need to strengthen postgraduate medical training and to educate doctors in line with the most up-to-date knowledge about vaccinations to meet patients’ expectations. For example, in Poland, all physicians involved in the immunization system have been equipped with educational materials on vaccines. Public authorities established (National Institute of Public Health) a dedicated internet platform (www.szczepienia.info [8]) which is a source of scientific-based knowledge about vaccination for both patients and physicians [29].

In response to the increasing activity of the anti-vaccination movement, nationwide educational campaigns on vaccinations were conducted by the Chief Sanitary Inspectorate—a public administration authority involved in public health services in Poland [30]. In addition to traditional TV interviews and expert debates, social media was a leading communication channel in the field of vaccine education. Moreover, in 2018, when the public bill was proceeded by the Polish Parliament, Chief Sanitary Inspector and Minister of National Education prepared an information brochure about vaccination, which was sent to all parents of school children (4.6 million students from nearly 24,000 schools from all over Poland) via an electronic journal (monitoring system of the child’s progress in learning) [30]. The increased confidence in vaccines and their safety in Poland in recent years may result from the nationwide debate about vaccines in mass media as well as communicational activities (focused on health promotion) carried out by the public health authorities using social media. Public health authorities should use novel technologies and social media to address new public health challenges. The current outbreak of novel coronavirus (SARS-CoV-2) and the media hype surrounding this can provide a good basis for discussions on infectious diseases and the need for preventive vaccinations.

There are different immunization systems within the European Union. In most Central and Eastern European countries including Poland, the Czech Republic, Slovakia, Slovenia, Hungary vaccinations are mandatory [10]. There are a growing number of EU countries that have expanded theirmandatory vaccination programmes [31,32]. In 2017, Italy extended the number of compulsory vaccines from four to ten, which results in increase vaccine coverage for all vaccines [31]. In 2018, France extended its mandatory vaccination programme from three to eleven diseases [32]. This indicates that in Europe there is a tendency to extend the mandatory immunization system rather than to abolish the obligation. In our study, 8.5% of parents would stop vaccinating their children if the vaccination obligation was abolished. Changing the immunization system from mandatory to voluntary may lead to a loss of herd immunity. Between 2000 and 2017, the coverage of the first dose of a measles-containing vaccine has decreased in 12 EU member states [19]. Moreover, between 2017 and 2018, the number of refusals to vaccinate for non-medical reasons increased by one-third [19]. Epidemiological data showed that there is a growing number of primary immunodeficiency diseases [33]. Low vaccine rates may lead to a loss of herd immunity and the outbreak of contagious diseases among those populations who cannot be vaccinated because of health conditions [34]. The coronavirus disease (COVID-19) pandemic may have a significant impact on attitudes towards vaccination [35]. In the 21st century, the COVID-19 pandemic shows how quickly infectious diseases can spread around the world, and the number of new cases and deaths reported worldwide indicates that more and more people see the effects of an infectious disease. Lessons learned from the smallpox epidemic in Wrocław, Poland in 1963 showed that massive vaccination campaigns during the epidemic may be an effective tool to increase social awareness about the vaccination [36]. This emphasizes the need to use social media as a public health promotion channel, which allows the provision of scientific knowledge about vaccination to different social groups.

This study has several limitations. Firstly, vaccine confidence was assessed based on one key-question related to vaccine safety. Different questions can be used to assess vaccine safety including the most recognized—the WHO “3Cs” model. We cannot exclude that another definition of vaccine confidence may result in slightly different results. Nevertheless, undermining vaccination safety was the main activity of the anti-vaccination movement in Poland. Therefore, we considered the question about vaccine safety as crucial in the context of the vaccine confidence in Poland. Moreover, our study provides detailed characteristics of individuals who declared that they would stop vaccinating children when vaccination obligation is abolished. This observation is the unique value of our study. Secondly, we cannot exclude non-response bias. Thirdly, computer-assisted web interviewing (CAWI) research method excludes the possibility of interaction with the respondent (and hence the inability to assess the competences of the respondents, i.e., whether they sufficiently understand the questions asked). This research method includes only these subjects, who have an internet access. Nevertheless, 84% households in Poland have Internet access [37]. Most people become parents aged 25–35, and this is a group that is fluent in the Internet. Social media and websites are the main communication channel for the anti-vaccination movement in Poland, which justifies the choice of the CAWI research method.

## 5. Conclusions

This study demonstrates relatively high confidence in mandatory vaccination among adults in Poland. A significant correlation between vaccine confidence and trust in medicine and scientific knowledge points to the important role of healthcare professionals in strengthening public awareness about vaccinations. While most of society trusts in vaccine safety, young adults are the least trustful of vaccinations. Different communication channels including social media and web television may be needed to reach a group of vaccination skeptics. In the face of the wide activity of the anti-vaccination movement, changing the immunization system from compulsory to voluntary carries significant threats to public health and may lead to a loss of herd immunity.

## Figures and Tables

**Table 1:** Structure of the study participants by state of vaccine confidence (*n* = 1079).

Variable	Total *n* (%)	Mandatory Vaccinations (Vaccines) Are Safe					
		Strongly Disagree	Tend to Disagree	Hard to Say/Undecided	Tend to Agree	Strongly Agree	*p*
		% (95%CI)					
Overall	1079 (100)	3.4 (2.5–4.7)	4.5 (3.5–6.0)	17.5 (15.4–19.9)	35.9 (33.1–38.8)	38.7 (35.8–41.6)	
Gender							
Male	500 (46.3)	3.4 (2.1–5.4)	4.8 (3.2–7.0)	19.2 (15.9–22.9)	32.2 (29.2–37.4)	39.4 (35.2–43.7)	0.05
Female	579 (53.7)	3.4 (2.2–5.3)	4.3 (2.9–6.3)	16.1 (13.3–19.3)	38.2 (34.3–42.2)	38.0 (34.1–42.0)	
Age (years)							
18–24	134 (12.4)	2.2 (0.8–6.4)	6.0 (3.1–11.3)	14.2 (9.3–21.1)	36.6 (28.9–45.0)	41.0 (33.1–49.5)	0.04
25–34	233 (21.6)	6.0 (3.6–9.8)	6.0 (3.6–9.8)	24.5 (19.4–30.4)	28.8 (23.3–34.9)	34.8 (28.9–41.1)	
35–44	174 (16.1)	1.7 (0.6–4.9)	3.5 (1.6–7.3)	17.2 (12.3–23.5)	39.7 (32.7–47.1)	37.9 (31.1–45.3)	
45–54	200 (18.6)	4.5 (2.4–8.3)	5.0 (2.7–8.9)	17.0 (12.4–22.8)	36.0 (29.7–42.9)	37.5 (31.1–44.4)	
55 and over	338 (31.3)	2.4 (1.2–4.6)	3.3 (1.8–5.7)	14.5 (11.1–18.7)	38.5 (33.4–43.8)	41.4 (36.3–46.7)	
Marital status							
Single	186 (17.2)	3.8 (1.8–7.6)	5.5 (2.9–9.6)	23.2 (17.6–29.7)	36.0 (29.5–43.1)	31.7 (25.5–38.7)	0.5
Married	586 (54.3)	3.8 (2.5–5.6)	4.6 (3.2–6.6)	17.6 (14.7–20.9)	36.4 (32.6–40.3)	37.7 (33.9–41.7)	
Informal relationship	202 (18.7)	3.0 (1.4–6.3)	4.5 (2.4–8.3)	14.4 (10.2–19.9)	31.7 (25.7–38.4)	46.5 (39.8–53.4)	
Divorced	62 (5.8)	1.6 (0.3–8.6)	3.2 (0.9–11.0)	12.9 (6.7–23.5)	41.9 (30.5–54.3)	40.3 (29.0–52.8)	
Widowed	43 (4.0)	2.3 (0.4–12.1)	2.3 (0.4–12.1)	14.0 (6.6–27.3)	39.5 (26.4–54.4)	41.9 (28.4–56.7)	
Having children							
Yes	755 (70.0)	3.4 (2.4–5.0)	4.5(3.2–6.2)	16.7 (14.2–19.5)	36.6 (33.2–40.0)	38.8 (35.4–42.3)	0.9
No	324 (30.0)	3.4 (1.9–6.4)	4.6 (2.8–7.5)	19.4 (15.5–24.1)	34.3 (29.3–39.6)	38.3 (33.2–43.7)	
Educational level							
Primary	35 (3.2)	2.9 (0.5–1.5)	0.0 (0.0–9.9)	37.1 (23.2–53.7)	34.3 (20.8–50.9)	25.7 (14.2–42.1)	0.05
Vocational	98 (9.1)	1.0 (0.2–5.6)	3.1 (1.1–8.6)	19.4 (12.8–28.3)	38.8 (29.7–48.7)	37.8 (28.8–47.6)	
Secondary	513 (47.5)	2.5 (1.5–4.3)	4.9 (3.3–7.1)	18.1 (15.0–21.7)	36.3 (32.2–40.5)	38.2 (34.1–42.5)	
Higher	433 (40.1)	5.1 (3.4–7.6)	4.9 (3.2–7.3)	14.8 (11.8–18.4)	34.9 (30.5–39.5)	40.4 (35.9–45.1)	
Occupational status							
Active	690 (64.0)	3.2 (2.1–4.8)	5.5 (4.0–7.5)	17.0 (14.3–19.9)	24.8 (31.1–38.4)	39.6 (36.0–43.3)	0.2
Passive	389 (36.0)	3.9 (2.4–6.3)	2.8 (1.6–5.3)	18.5 (15.0–22.7)	37.8 (33.1–42.7)	37.0 (32.4–41.9)	
Professional activity							
Employee	625 (57.9)	3.5 (2.3–5.3)	5.3 (3.8–7.3)	17.0 (14.2–20.1)	34.9 (31.3–38.7)	39.4 (35.6–43.2)	0.1
Self-employed	65 (6.0)	0.0 (0.0–5.6)	7.7 (3.3–16.8)	16.9 (9.7–27.8)	33.9 (23.5–46.0)	41.5 (30.4–53.7)	
Unemployed	111 (10.3)	2.7 (0.9–7.7)	3.6 (1.4–8.9)	28.8 (21.2–37.9)	35.1 (26.9–44.4)	29.7 (22.0–38.8)	
Retired	224 (20.8)	3.6 (1.8–6.9)	2.7 (1.2–5.7)	14.7 (10.7–19.9)	39.3 (33.1–45.8)	39.7 (33.6–46.3)	
Student	54 (5.0)	7.4 (2.9–17.6)	1.9 (0.3–9.8)	13.0 (6.4–24.4)	37.0 (25.4–50.4)	40.7 (28.7–54.0)	
Place of residence							
Rural	330 (30.6)	3.3 (1.9–5.9)	3.9 (2.3–6.6)	18.5 (14.7–23.0)	35.8 (30.8–41.1)	38.5 (33.4–43.8)	0.5
City up to 20,000 residents	135 (12.5)	5.2 (2.5–10.3)	5.9 (3.0–11.3)	20.7 (14.8–28.3)	29.6 (22.6–37.8)	38.5 (30.7–46.9)	
City between 20,000–100,000 Residents	226 (21.0)	2.2 (0.9–5.1)	7.1 (4.4–11.2)	17.7 (13.3–23.2)	37.2 (31.1–43.6)	35.8 (29.9–42.3)	
City between 100,000–500,000 Residents	229 (21.2)	4.4 (2.4–7.9)	3.1 (1.5–6.2)	17.0 (12.7–22.4)	37.6 (31.5–43.9)	38.0 (31.9–44.4)	
City above 500,000 residents	159 (14.7)	2.5 (0.9–6.3)	3.1 (1.3–7.2)	13.2 (8.8–19.4)	37.1 (29.9–44.8)	44.0 (36.5–51.8)	

**Table 2:** Socioeconomic factors associated with vaccine confidence (*n* = 1079).

	Mandatory Vaccinations (Vaccines) Are Safe					
Variable	Yes		Univariate Logistic Regression	Multivariate Logistic Regression		
				Model 1	Model 2	Model 3
	*n*	% (95%CI)	OR (95%CI)	aOR (95%CI)		
Gender						
Male	363	72.6 (68.5–76.3)	Ref.	Ref.	Ref.	Ref.
Female	441	76.2 (72.5–79.5)	1.21 (0.92–1.59)	1.19 (0.89–1.57)	1.20 (0.90–1.60)	1.20 (0.91–1.60)
Age (years)						
18–24	104	77.6 (69.8–83.8)	Ref.	Ref.	Ref.	Ref.
25–34	148	63.5 (57.2–69.4)	0.50 (0.31–0.82)*	0.52 (0.32–0.86)*	0.48 (0.29–0.80)*	0.48 (0.29–0.80)*
35–44	135	77.6 (70.8–83.2)	1.00 (0.58–1.71)	1.06 (0.60–1.87)	0.97 (0.54–1.74)	0.96 (0.54–1.72)
45–54	147	73.5 (67.0–79.1)	0.80 (0.48–1.34)	0.86 (0.49–1.50)	0.80 (0.45–1.40)	0.79 (0.45–1.40)
55 and over	270	79.9 (75.3–83.8)	1.15 (0.71–1.86)	1.21 (0.71–2.05)	1.22 (0.71–2.10)	1.24 (0.72–2.13)
Marital status						
Never married	370	75.1 (71.1–78.7)	Ref.	Ref.	Ref.	Ref.
Ever married	434	74.1 (70.4–77.5)	0.95 (0.72–1.25)	0.85 (0.61–1.18)	0.83 (0.60–1.16)	0.83 (0.59–1.15)
Having children						
No	235	72.5 (67.4–77.1)	Ref.	Ref.	Ref.	Ref.
Yes	569	75.4 (72.2–78.3)	1.16 (0.86–1.56)	1.06 (0.73–1.53)	1.05 (0.73–1.52)	1.06 (0.73–1.53)
Educational level						
Less than higher	478	74.0 (70.5–77.2)	Ref.	-	Ref.	Ref.
Higher education	326	75.3 (71.0–79.1)	1.07 (0.81–1.42)	-	1.09 (0.82–1.46)	1.10 (0.82–1.48)
Occupational status						
Passive	291	74.8 (70.3–78.9)	Ref.	-	Ref.	Ref.
Active	513	74.4 (71.0–77.5)	0.98 (0.73–1.30)	-	1.24 (0.89–1.71)	1.24 (0.90–1.72)
Place of residence						
Urban	559	74.6 (71.4–77.6)	Ref.	-	-	Ref.
Rural	245	74.2 (69.3–78.7)	0.98 (0.73–1.32)	-	-	1.09 (0.80–1.48)

OR–odds ratio; aOR–adjusted odds ratio; * *p*< 0.05.

**Table 3:** Correlations between vaccine confidence and trust in doctors and medicine, scientific knowledge and the government (*n* = 1079).

		Mandatory Vaccinations (Vaccines) Are Safe	Doctors can be Trusted	I Trust My Doctor	Only Scientific Knowledge Is Trustworthy	Horoscopes Contain a lot of Truth	Governments usually Want the Well-Being of Citizens
Mandatory vaccinations (vaccines) are safe	r	-					
	*p*						
Doctors can be trusted	r	0.33	-				
	*p*	0.0001					
I trust my doctor	r	0.35	0.62	-			
	*p*	0.0001	0.0001				
Only scientific knowledge is trustworthy	r	0.19	0.27	0.22	-		
	*p*	0.0001	0.0001	0.0001		-	-
Horoscopes contain a lot of truth	r	−0.13	0.06	−0.02	0.08	-	-
	*p*	0.0001	0.07	0.6	0.01		
Governments usually want the well-being of citizens	r	−0.02	0.24	0.13	0.15	0.18	-
	*p*	0.6	0.0001	0.0001	0.0001	0.0001	

r—The Spearman rank correlation coefficient.

**Table 4:** Socioeconomic factors associated with discontinuation of children vaccination among parents (*n* = 738).

		Mandatory Vaccinations (Vaccines) Are Safe							
Variable	Total	Yes		Univariate Logistic Regression	Multivariate Logistic Regression				
					Model 1		Model 2		Model 3
	*n* (%)	*n*	% (95%CI)	OR (95%CI)	aOR (95%CI)				
Gender									
Male	300 (40.6)	20	6.7 (4.4–10.1)	Ref.	Ref.	Ref.		Ref.	
Female	438 (59.4)	43	9.8 (7.4–13.0)	1.53 (0.88–2.65)	1.51 (0.86–2.64)	1.49 (0.85–2.63)		1.51 (0.86–2.66)	
Age (years)									
18–34	160 (21.7)	17	10.6 (6.7–16.4)	Ref.	Ref.	Ref.		Ref.	
35–54	291 (39.4)	23	7.9 (5.3–11.6)	0.73 (0.38–1.42)	0.74 (0.38–1.45)	0.75 (0.38–1.48)		0.77 (0.39–1.52)	
55 and over	287 (38.9)	23	8.0 (5.4–11.7)	0.72 (0.37–1.40)	0.76 (0.39–1.47)	0.67 (0.33–1.35)		0.61 (0.30–1.25)	
Marital status									
Never married	219 (29.7)	16	7.3 (4.6–11.5)	Ref.	Ref.	Ref.		Ref.	
Ever married	519 (70.3)	47	9.1 (6.9–11.8)	1.26 (0.70–2.28)	1.35 (0.74–2.46)	1.36 (0.75–2.48)		1.49 (0.81–2.74)	
Educational level									
Less than higher	428 (58.0)	32	7.5 (5.4–10.4)	Ref.		Ref.		Ref.	
Higher education	310 (42.0)	31	10.0 (7.1–13.8)	1.38 (0.82–2.31)		1.46 (0.86–2.48)		1.37 (0.81–2.33)	
Occupational status									
Passive	260 (35.2)	25	9.6 (6.6–13.8)	Ref.		Ref.		Ref.	
Active	478 (64.8)	38	8.0 (5.9–10.7)	0.81 (0.48–1.38)		0.73 (0.40–1.33)		0.70 (0.39–1.29)	
Place of residence									
Urban	520 (70.5)	50	9.6 (7.4–12.5)	Ref.				Ref.	
Rural	218 (29.5)	13	6.0 (3.5–9.9)	0.60 (0.32–1.12)				0.56 (0.29–1.07)	

OR–odds ratio; aOR–adjusted odds ratio.
